# Neonatal Seizures and Purinergic Signalling

**DOI:** 10.3390/ijms21217832

**Published:** 2020-10-22

**Authors:** Aida Menéndez Méndez, Jonathon Smith, Tobias Engel

**Affiliations:** 1 Department of Physiology and Medical Physics, RCSI University of Medicine and Health Sciences, Dublin D02 YN77, Ireland; aidammendez@rcsi.ie (A.M.M.); jonathonsmith@rcsi.ie (J.S.); 2 FutureNeuro, Science Foundation Ireland Research Centre for Chronic and Rare Neurological Diseases, RCSI University of Medicine and Health Sciences, Dublin D02 YN77, Ireland

**Keywords:** neonatal seizures, development, ATP, purinergic signalling, P2X7 receptor

## Abstract

Neonatal seizures are one of the most common comorbidities of neonatal encephalopathy, with seizures aggravating acute injury and clinical outcomes. Current treatment can control early life seizures; however, a high level of pharmacoresistance remains among infants, with increasing evidence suggesting current anti-seizure medication potentiating brain damage. This emphasises the need to develop safer therapeutic strategies with a different mechanism of action. The purinergic system, characterised by the use of adenosine triphosphate and its metabolites as signalling molecules, consists of the membrane-bound P1 and P2 purinoreceptors and proteins to modulate extracellular purine nucleotides and nucleoside levels. Targeting this system is proving successful at treating many disorders and diseases of the central nervous system, including epilepsy. Mounting evidence demonstrates that drugs targeting the purinergic system provide both convulsive and anticonvulsive effects. With components of the purinergic signalling system being widely expressed during brain development, emerging evidence suggests that purinergic signalling contributes to neonatal seizures. In this review, we first provide an overview on neonatal seizure pathology and purinergic signalling during brain development. We then describe in detail recent evidence demonstrating a role for purinergic signalling during neonatal seizures and discuss possible purine-based avenues for seizure suppression in neonates.

## 1. Introduction

Neonatal seizures are a clinical emergency affecting 3–5 out every 1000 live births and are one of the most common comorbidities of neonatal encephalopathy, with seizures aggravating acute injury and clinical outcomes [1,2]. Neonatal seizures result in a mortality rate up to 20% and contribute to long-term outcomes including epilepsy, cerebral palsy, developmental delay and psychomotor deficits [3,4]. Current treatment strategies aim to reduce the hyperexcitability of brain tissue via the use of anti-seizure drugs (ASDs), with phenobarbital being the first-line drug for neonatal seizures. ASDs, however, fail to resolve seizures in 50% of infants and may exacerbate symptoms and later life neurological deficits [2,5]. Therefore, there is a pressing need to identify novel treatment options with higher response rates and without affecting normal development of the brain.

Purinergic signalling refers to the extracellular communication between cells mediated via purine nucleotides and nucleosides, such as adenosine triphosphate (ATP) and adenosine. The purinergic system involves a complex regulatory machinery including regulatory proteins of purine release and uptake, cell membrane receptors and metabolizing enzymes to remove purines from the extracellular space [6,7]. Research over the past decades has demonstrated purinergic signalling to be involved in literally all human pathological conditions ranging from bone diseases, cancer and diabetes to diseases of the central nervous system (CNS) [8]. In the CNS, targeting different components of the purinergic signalling cascade has been proposed as a potential treatment strategy for a range of different diseases including chronic neurodegenerative diseases (e.g., Alzheimer’s disease), psychiatric diseases (e.g., depression), neurological disease epilepsy and acute insults to the brain such as a stroke or traumatic brain injury [9,10,11]. Emerging evidence also suggests a role for purinergic signalling during early developmental disorders such as schizophrenia and autism spectrum [8,12]. Early brain development comprises a sequence of specific events including proliferation (neurogenesis/gliogenesis), differentiation, migration of neuronal precursors, neuronal network formation and synaptogenesis. Critically, purinergic signalling has been shown to be involved in all of these processes [13]. More recent data now also suggests purinergic signalling to be involved during acute insults to the immature brain including neonatal seizures [14].

In this review, we first provide a summary of neonatal seizures, including current treatments and animal models for its study. We then summarize the different elements of the purinergic system and its role during CNS development. Finally, we discuss current knowledge regarding the role of purinergic signalling during neonatal seizures and provide potential directions for future research.

## 2. Neonatal Seizures

Seizures are a period of excessive and highly synchronous neuronal brain activity and are one of the most common neurological disorders in newborns admitted to the intensive care unit [2]. Normally, seizures are indicative of an underlying dysfunction in the brain. Early life seizures are widely described as a neurological emergency due to a mortality rate as high as 23% and are well documented to cause later life comorbidities such as postnatal epilepsy and global neurodevelopmental delay [3,15]. A seizure is presented when the physiology of the brain abnormally favours excitatory neurotransmission, i.e., promotion of glutamatergic and disinhibition of γ-aminobutyric acid (GABA)ergic transmission. The neonatal brain is in a hyperexcitable state, essential for normal brain development including processes such as synaptogenesis, dendritic spine density development, glial proliferation, myelination and axon guidance [16,17]. Unfortunately, this hyperexcitable state renders the neonatal population at a greater risk to develop seizures particularly within the first two days of life [18,19]. In fact, the incidence rate of seizures in neonates is between 1.8–3.5 per 1000 live births and 10-fold higher in pre-terms [20,21]. Furthermore, any interference, such as a seizure, during these critical neurodevelopmental mechanisms may produce serious consequences persisting into adulthood. For example, early elevated inflammation is associated with network reorganisation with the potential for epileptogenic circuits and psychiatric disorders, as seen in animal models [16,22,23,24]. Depending on the study, 20–50% of seizure survivors will express some form of neurodevelopment disability in later life [3,25]. In fact, a comprehensive review of studies which evaluated an overall population of 4538 newborns with neonatal seizures observed that 17.9% developed postneonatal epilepsy [26].

### 2.1. Aetiologies of Neonatal Seizures

Presentation of neonatal seizures is most commonly symptomatic of an underlying aetiology rather than idiopathic. Many risk factors associated with neonatal seizures are related to a metabolic imbalance during pregnancy or immediately postdelivery, including perinatal infection, hypoglycaemia and intracranial haemorrhage [4,15,27]. Moreover, rare cases of an inborn genetic component of neonatal seizures exist, with the majority altering metabolic pathways, including KCNQ2 mutations, infantile hypophosphatasia (mutations in the tissue nonspecific alkaline phosphatase (TNAP)) and propionic acidaemia (deficiency of propionyl-CoA carboxylase) [27]. However, the most common aetiologies of neonatal seizures are acute neurological insults to the brain that limit oxygen and glucose delivery. This includes ischemic stroke; intracranial haemorrhage; and the most common cause, accounting for 40–60% of neonatal seizure cases, hypoxic-ischemic encephalopathy (HIE) [28,29,30]. Birth asphyxia, that precedes HIE, is the third most common cause of neonatal mortality (23%), behind infection (36%) and preterm births (28%) [31]. HIE is caused by events that limit efficient oxygen delivery to the preterm or neonatal brain tissue, such as foetal distress or placental pathology. However, neonatal seizures are only presented in moderate or severe HIE [32,33]. Neonatal seizure aetiology can be difficult to determine, with the timing of the first seizure normally a good indicator. In line with this, HIE-induced seizures usually present within the first 48 h of life with the other aetiologies having a later seizure onset [34].

### 2.2. Animal Models of Neonatal Seizures

Clinical investigation can provide information on aetiologies and consequences of neonatal seizures; however, animal studies are a requirement to elucidate pathogenic mechanisms and possible novel treatments. Many animal models of neonatal seizures are derivatives of adult seizure models. This is typical of models where a chemoconvulsant (e.g., kainic acid (KA), pentylenetetrazole (PTZ) or flurothyl) is used to trigger seizures [35,36,37,38]. Direct delivery into the brain of KA, a glutamate receptor agonist, to illicit seizures was first achieved by Ben Ari et al. in 1978 [39]. This model can be translated for use in neonatal rats (P10), in which Mesuret et al. microinjected KA into the amygdala to illicit electrographic nonterminating seizures that persist for at least 1 h, with hippocampal neuronal damage observed 72 h later [40]. An intraperitoneal injection of PTZ, a GABA_A_ receptor antagonist, can also induce neonatal seizures at any age; however, the pattern of seizures and dose required is age-dependent [41]. It is also possible to illicit seizures with multiple low doses of PTZ [42]. These models are widely used to screen preclinical and currently available drugs at various ages ranging from neonatal to adult [43]. PTZ-induced neonatal seizures at P10 in rats produced neuronal damage yet not neuronal death, a feature common among neonatal seizure models [44]. Despite these models not encompassing a translatable seizure induction to the clinic, they are extremely useful in investigating seizure pathophysiology. However, before stark conclusions can be made, results must be validated in a model more similar to the human condition.

With limited oxygen and glucose delivery predominately responsible for most neonatal seizure cases, many experimental models are built to recapitulate clinical features of HIE and subsequent seizures. The Rice–Vannucci model, first published in 1981, was first to encapsulate features of neonatal ischemia and is the basis of current animal models in which hypoxia-ischemia induces neonatal seizures [45]. This model involves ligating the common carotid artery unilaterally (MCAO (medial carotid artery occlusion)), followed by a brief period of hypoxia in neonatal rats (P7). This was developed from a previous model of hypoxia ischemia in adult rats [46]. Further reiterations of the Rice–Vannucci model have been utilised that vary in the degree of the hypoxia insult (8% O_2_, for 30 min–2.5 h), the species of rodent and the age of the rodent used (P2—adulthood). This model is primarily used to study HIE. However, using video-electroencephalogram (EEG), Cuaycong et al. validated this model for use in neonatal seizures in which a period of 90 min hypoxic (8% O_2_) insult is required to illicit acute seizures in P10–12 rats [47]. Kadam et al. also observed epileptogenesis in this model (P7 rats, MCAO and 8% O_2_ for 2 h), with 56% of rats developing spontaneous seizures in later life [48]. The age of the rodent is an important consideration to make due to the neurodevelopment of the rodent occurring rapidly. P7 age is widely used as it relates to the same brain maturation state as 36-week gestation in a human infant, the final week of gestation, with P10 representing a term infant [49].

More recently, mice have been utilized for neonatal seizure studies by using either a combination of MCAO and hypoxia [50] or hypoxia alone [51,52]. In 2015, Rodriguez et al., building upon these studies, developed a noninvasive model of global hypoxia in mice [53]. Briefly, P7 mice were subjected to 15 min of hypoxic conditions (5% O_2_) and presented with symptomatic seizures during at least 1 h post-hypoxia. When assaying other ages, mice either had high mortality or did not present with seizures, highlighting how the age of mice must be carefully considered. This model also encapsulates post-seizure morbidity, with mice who underwent infantile hypoxia showing an increased seizure susceptibility and development of multiple behavioural deficits in later life. These studies invite the use of transgenic mouse lines in neonatal seizure studies. This could add great power by dissecting the complex network of pathophysiological systems following a neonatal insult.

### 2.3. Current Treatment for Neonatal Seizures

Treatment for neonatal seizures with a known genetic or metabolic component can be relatively simple. For example, seizures attributed to a mutation in the TNAP gene resulting in hypophosphatasia, a deficiency in vitamin B6 metabolism, can be controlled with pyridoxine, the phosphorylated form of vitamin B6 [54,55]. Acute symptomatic seizures, such as those following HIE in infants, have proven much harder to treat. The current standard of care for HIE is to initiate therapeutic hypothermia, and if neonatal seizures are present, a course of at least one anti-seizure medication as well [56,57]. Therapeutic hypothermia has proved very successful to reduce the acute seizure burden and mortality following HIE [58,59,60]. Unfortunately, therapeutic hypothermia is only effective to reduce the seizure burden in moderate HIE cases and not in severe cases [59]. Therapeutic hypothermia’s ability to prevent the later life comorbidities remains inconclusive due to the limited number of studies investigating this. Rates of developing cerebral palsy and neurodevelopmental delay were reduced following therapeutic hypothermia when examined at 18–22 months [60]; however, no significant conclusion regarding therapeutic hypothermia’s ability to prevent disability could be made when followed up in later life [61].

For acquired seizures not initiated by HIE, currently, the only treatment strategy is anti-seizure medications, which act to inhibit excitatory glutamatergic or promote GABAergic neurotransmission. These medications are useful and certainly are effective in many cases, yet a level of pharmacoresistance remains, particularly in symptomatic neonatal seizures [62]. Also, concerns have been raised with safety of anti-seizure medications in the developing brain. The three most popular anti-seizure drugs, phenobarbital, valproate and phenytoin, that all act upon different neurotransmitter systems, have all been shown to induce apoptotic neurodegeneration in the developing rodent brain [63]. This can be attributed to the developmental expression levels of these neurotransmitter systems, and hence, the onset of certain drug administration needs careful consideration. The first-line anti-seizure medication is phenobarbital, acting as a positive allosteric modulator of the GABA_A_ receptor. However, phenobarbital remains ineffective in around 50% of neonates to manage seizures [64]. In the immature brain, GABA_A_ activation leads to an efflux of chloride ions to promote excitatory neurotransmission needed for natural brain development [65,66]. Nevertheless, this makes the immature brain more susceptible to seizures and therapies targeting GABA may even potentiate seizures and excitotoxicity. There are multiple studies raising concern with phenobarbital’s safety due to potentiation of neuronal damage and behavioural deficits observed in rodent models [5,67,68] and various reports of patients developing behavioural abnormalities in later life [69]. In fact, Torolina et al. observed that phenobarbital and midazolam exacerbate neonatal seizure damage even at subclinical doses [68]. Due to the damage seen with current medications, careful consideration is needed to outweigh the risks of seizure management with the possibility to potentiate neuronal damage. Therefore, there is an urgent need to develop new treatments that act upon nonclassical mechanisms of seizure prevention with minimal impact on neurodevelopment. Furthermore, there is limited evidence of therapies to protect against long-term consequences of neonatal seizures, and as such, current clinical focus is targeting initial neonatal seizure [4,70]. In recent years, with better standard of care and earlier diagnosis for neonates, mortality rates have decreased, yet the levels of later life neurological sequelae remain unchanged [71,72], suggesting that current medications are not tackling this aspect effectively.

## 3. The Purinergic System

**Figure 1:**
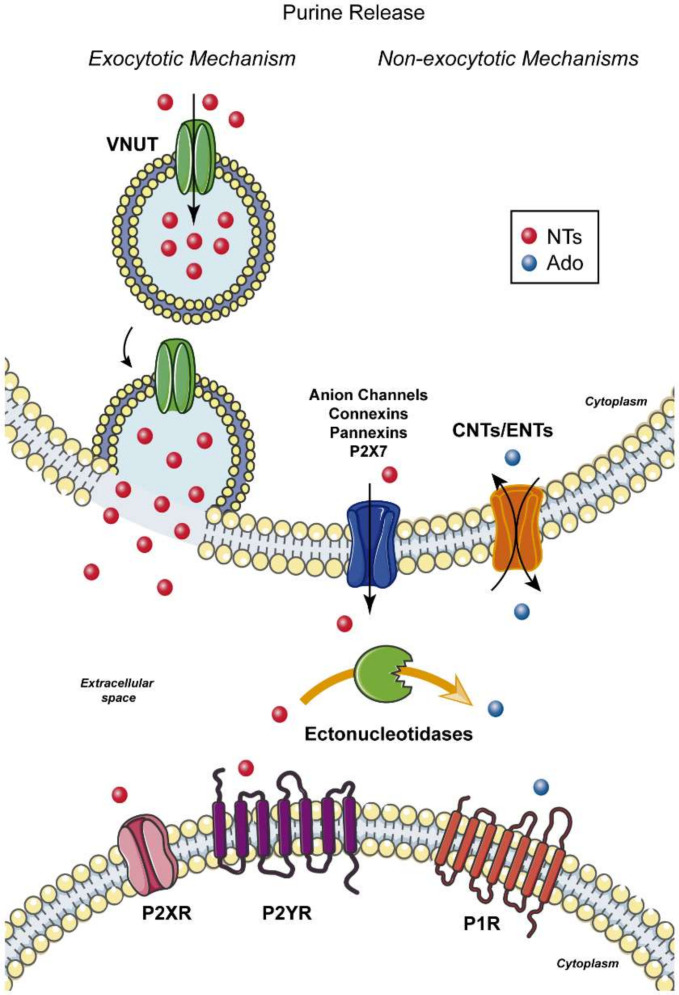
Purine release mechanisms: purines such as ATP and adenosine can be actively released from neurons and glial cells including microglia and astrocytes or passively from damaged or dying cells. Schematic showing the different release mechanisms including exocytotic and non-exocytotic mechanisms. Exocytotic mechanisms require previous storage of nucleotides via the vesicular nucleotide transporter (VNUT) in secretory/synaptic vesicles. Non-exocytotic mechanisms include the release of nucleotides by different types of channels, such as anion channels, pannexins and connexins. In contrast to ATP, adenosine can also be released into the extracellular space via Concentrative Nucleoside Transporters (CNTs) and Equilibrative Nucleoside Transporters (ENTS). Released nucleotides activate P2X and P2Y receptors localized on neuronal or glial membranes. Simultaneously, the hydrolysis of nucleotides by ectonucleotidases produces adenosine which, in turn, activates P1 receptors. Abbreviations: NTs, nucleotides; Ado, adenosine; VNUT, vesicular nucleotide transporter; CNTs, Concentrative Nucleoside Transporters; ENTs, Equilibrative Nucleoside Transporters.

Purinergic signalling represents probably one of the most ancient cellular signalling systems. Accordingly, purinergic signalling is an essential signalling system employed by the majority of cells across species with key roles during health and disease [73]. Purinergic signalling comprises a complex regulatory system including nucleoside and nucleotide channels and transporters, purinergic receptors, ectonucleotide-metabolizing enzymes and ectonucleoside transporters [10] (Figure 1). The particular high expression of different components of the purinergic system within the CNS highlights its importance in normal brain function. As such, purinergic signalling is involved in a plethora of different cellular pathways including synaptic transmission, in which purine nucleotides and nucleosides act as neuro- and gliotransmitters or modulators [74,75,76]; cell proliferation and differentiation [77,78]; mediation of communication between astrocytes and reciprocal communication between neurons and glia [79,80,81]; and inflammatory processes [82,83,84,85,86]. The following section will briefly introduce the different components of the purinergic system and highlight their relevance to normal brain function.

### 3.1. Purine Release

The release of ATP and other nucleotides and nucleoside including adenosine into the extracellular space occurs via different mechanisms depending on cell type and physiological context. Non-exocytotic mechanisms include anion channels, such as plasmalemma voltage-dependent anion channels [87]; ATP-binding cassette transporters, such as the cystic fibrosis transmembrane conductance regulator Cl^−^ channel [88]; the purinergic P2X7 receptor [89,90]; and hemichannels, including connexin-43 [91] and pannexins [92,93]. The pannexin family comprises three members: Pannexin 1 (Panx1), Panx2 and Panx3 [94]. Among members of this family, Panx1 is the only one which forms functional channels and is expressed in both neuronal and glial cells in the brain [95,96]. Panx1 can be activated by different mechanisms including depolarization, mechanical stress or elevated intracellular Ca^2+^ concentrations [97,98,99,100]. Moreover, Panx1 may also contribute to ATP release after P2X7 activation, suggesting a direct connection between P2X7 and Panx1 [93,101]. Release of ATP and other nucleotides via exocytosis in the CNS has been reported from several cell types including neurons [102,103], astrocytes [104] and microglia [105]. Finally, the Cl^−^-dependent vesicular nucleotide transporter (VNUT) has been described to mediate the storage of ATP and other nucleotides in secretory and synaptic vesicles [106]. This transporter is highly expressed in different brain regions including the olfactory bulb, hippocampus and cerebellum [103] and has been shown to be functional in different types of neurons [102,107,108,109] and populations of glial cells [104,105,109].

### 3.2. The Purinergic Receptor Family

Nucleotides and nucleosides activate a large number of different cell-surface receptors divided into two major families termed purinergic P1 and P2 receptors. Whereas P1 receptors respond to adenosine and adenosine mono-phosphate (AMP), P2 receptors can be activated by ATP, adenosine diphospate (ADP), uridine triphospate (UTP), uridine monophosphate (UDP), nucleotide sugars, dinucleoside polyphosphates and NAD^+^ [75].

#### 3.2.1. P1 Receptor Family

P1 receptors are G protein-coupled and include four isoforms: A1, A2_A_, A2_B_ and A3 receptors. While in general A2_A_ and A2_B_ receptors induce the production of cyclic AMP (cAMP) via the Gs family, A1 and A3 receptors are usually coupled to Gi/o proteins, thereby inhibiting the production of cAMP. Other G protein combinations have, however, been described [110,111]. In the CNS, adenosine plays several roles, such as the modulation of neural and glial functions, neuron-glia signalling, neural development and the control of neurotransmitter release [112,113,114,115,116], with adenosine receptors expressed in both neurons and glia (astrocytes and microglia). Among the different P1 receptor subtypes, A1 and A2_B_ receptors are usually associated with physiological neuronal processes (e.g., control of neurotransmitters release [117,118]) whereas A2_A_ and A3 receptors are thought to be mostly activated under pathological conditions (e.g., epilepsy, neuropathy, neurodegenerative disorders or psychiatric conditions [119,120,121,122]). Because the dysregulation of the adenosinergic system is implicated in different pathologies, several studies have focused on this system as an avenue for new treatments. While A2_A_ inhibition has shown neuroprotective properties during clinical trials in patients with Parkinson’s [123], the activation of A1 receptors has been shown to reduce chronic pain [124] and to protect against epilepsy [125] and cerebral ischemia [126].

#### 3.2.2. P2 Receptor Family

Among the P1 receptors, the A1 receptor subtype presents the most extensive distribution in the CNS, including limbic and neocortical brain regions, basal ganglia, brainstem, diencephalon and cerebellum. Regarding its cell type-specific expression, A1 receptor subtypes have been shown to be expressed in neurons, astrocytes, oligodendrocytes and microglia. A1 receptors, via Gi and Go interaction, inhibit adenyl cyclase with the subsequent decrease of cAMP levels, reduction of protein kinase A activation and inhibition of GABA uptake into astrocytes [127,128]. A1 receptor activation has been linked to several pathological conditions including neurodegeneration, pain and seizures [129,130,131,132,133]. Counteracting increased hyperexcitability states in the brain, A1 receptors mediate the inhibition of *N*-type calcium channels and the activation of G protein-coupled inwardly rectifying potassium channels [134,135], block presynaptic glutamate release and decrease the activation of the postsynaptic glutamate receptor *N*-methyl-d-aspartate receptor (NMDA), resulting in the suppression of neuronal activity [136,137]. The expression of A2_A_ receptors is mainly located at the postsynaptic region of the encephalin-containing medium spiny neurons of the indirect pathway of the basal ganglia. A2_A_ receptors are related to the activation of adenylate cyclase [138] through its coupling with Gs proteins. A2_A_ receptor activation promotes the increase of NMDA receptor function and glutamate release at glutamatergic axon terminals [139,140,141,142]. Similar to A2_A_ receptors, A2_B_ receptors also activate adenylate cyclase and are ubiquitously expressed in the brain. However, there is not a clear link between these receptors and physiological or behavioural responses most likely due to the lack of specific agonists or antagonists. Finally, A3 receptors can uncouple A1 receptors and decrease thereby their inhibitory effects [143] via a protein kinase C-dependent mechanism. Although the presence of A3 receptors is low in the brain [144], its expression has been detected in hippocampal neurons [143], astrocytes [145] and microglial cells [146].

P2 receptors are subdivided into two subfamilies according to mechanism of action, pharmacology and molecular cloning, including the fast-acting P2X ligand-gated ion channels [147,148] and the slower-acting G-protein coupled P2Y receptors [149,150,151]. P2 receptors diverge in their molecular properties, amino acid sequences and relative sensitivities to ATP (e.g., nanomolar (P2Y receptors), low micromolar (most P2X receptors) and high micromolar (P2X7 receptor)). The structure of P2X receptors consists of two transmembrane domains: an intracellular *C*- and *N*- terminus and a large extracellular loop [147]. Most of the conserved regions are located in the extracellular loop, whereas transmembrane domains are less conserved between P2X receptors [152,153,154]. To date, seven mammalian subunits have been cloned (P2X1-7) [147] which form either functional homo- or heterotrimers exhibiting a high diversity due to the assembly of different individual subunits [155,156,157,158]. Among the P2X receptors, the P2X7 receptor has unique characteristics including the lowest affinity for ATP (approximately 100 µM) and a slower desensitization [159]. Functional expression of all P2X subunits has been shown within the brain on both neurons and glia [160]. Ionotropic P2X receptors, via the binding of extracellular ATP, open a permeable pore to the cations Na^+^, K^+^ and Ca^2+^. In the brain, P2X receptor activation is involved in the regulation of synaptic plasticity in different brain circuits and fast synaptic transmission [161,162]. Synaptic currents induced by P2X activation contribute only 5–15% to fast excitatory transmission, possibly due to their high Ca^2+^ permeability at hyperpolarized membrane potentials [151,163]. However, the contribution of P2X-mediated currents might be higher under pathological conditions, such as a seizure, by increasing the influx of Ca^2+^ and by elevating the release of neurotransmitters such as glutamate [164,165]. P2X receptors are involved in a multitude of Ca^2+^-sensitive processes including cellular proliferation, differentiation, maturation and survival, cell communication, migration and inflammation [166].

The metabotropic P2Y receptor family comprises eight G-protein coupled receptors: P2Y_1_, P2Y_2_, P2Y_4_, P2Y_6_, P2Y_11_, P2Y_12_, P2Y_13_ and P2Y_14_. All P2Y receptors share the topology of G-protein coupled receptors, which is characterised by seven transmembrane-domains, an extracellular amino and an intracellular carboxyl terminus. Moreover, P2Y receptors form homo- or heterodimers with other P2Y subunits [167] or with other receptors such as adenosine receptors [149]. Several P2Y receptors, including P2Y_1_, P2Y_2_, P2Y_4_, P2Y_6_ and P2Y_11_, are coupled to Gq/G_11_, which promotes endoplasmic reticulum Ca^2+^ release through the phospholipase C/inositol triphosphate pathway. P2Y_12_, P2Y_13_ and P2Y_14_ receptors are coupled to Gi/Go proteins which inhibit adenylyl cyclase, resulting in a decrease of cAMP production. The P2Y_11_ receptor is an exception as it is also able to couple to Gs, which stimulates adenylyl cyclase, thereby increasing cAMP production [150,168]. Depending on the P2Y receptor subtype, P2Y receptors can be activated by different nucleotides including ATP, ADP, UDP and sugar nucleotides [150,169,170,171,172]. Similar to P2X receptors, P2Y receptors are present at very early stages of embryonic CNS development [158] and are expressed on both neurons and glia, being involved in different processes such as modulation of neurotransmitter release [173,174], cell survival and neuroinflammation [175,176].

The purinergic system and neuroinflammation are tightly linked, with purinergic signalling described as fundamental for microglia’s physiological roles and proconvulsive cytokine release [177]. A diverse number of purinergic receptors is expressed on microglial cells, where they exert different effects. Activation of microglial A1_A_ receptors potentially removes microglia from a pro-inflammatory phenotype [178], with A2_A_ receptors critical for microglia process retraction [86]. Among P2X receptors, P2X7 is often portrayed as a key driver of pathological inflammation. P2X7 is widely expressed in microglia [179,180] and has been described as essential for the NLRP3 inflammasome activation and subsequent release of Interleukin-1β (IL-1β) [181]. The P2X4 receptor has a described role in microglia chemotaxis and activation [182,183]. Likewise, the activation of several P2Y receptors in microglia, such as P2Y_1_ and P2Y_12_, promotes its phagocytic activity, migration towards damaged region and the release of IL-1β [184,185,186,187]. Alves et al. showed the context-dependent role of the P2Y_1_ receptor to seizure pathology involving its expression in microglia [188]. Also, astrocytic P2Y_1_ has been described as responsible for the spread of neuronal hyperexcitability throughout the brain via mediating glutamate gliotransmission [189]. P2Y_12_ is expressed in microglia throughout its life cycle and again has prominent roles in microglia upregulation and migration [190]. With inflammation becoming increasing associated with seizure pathology [191], purinergic signalling will have major roles in mediating this.

### 3.3. Ectonucleotidases

Ectonucleotidases are enzymes with an extracellularly oriented catalytic site which rapidly hydrolyses ATP and other nucleotides after their release. These enzymes, operating in concert or consecutively, control the lifetime of extracellular released nucleotides by degrading or interconverting the originally released nucleotide generating ligands for additional P2 or P1 receptors. Ectonucleotidases comprise several families of enzymes divided by their functional and molecular properties including substrate specificity, product formation, optimal catalytic pH and cationic dependence [192].

All ectonucleotidase families are expressed in the brain including ectonucleoside triphosphate diphosphohydrolases (E-NTPDases/CD39), ectonucleotide pyrophosphatase and/or phosphodiesterases (E-NPPs), alkaline phosphatases and ecto-5′-nucleotidase [192]. The E-NTPDase/CD39 family comprises four surface-located members (E-NTPDase 1, 2, 3 and 8) which hydrolyse ATP into ADP or AMP, and ADP to AMP, exhibiting a different affinity for each nucleotide. E-NTPDase1 (also called CD39) presents equal affinity for ATP and ADP, whereas E-NTPDases 2, 3 and 8 are more selective for ATP [193]. The E-NPP consists of 7 enzymes (NPP1–7) which are able to cleave ATP directly into AMP [194]. Moreover, E-NPPs also hydrolyse dinucleoside polyphosphates and UDP sugars. AMP produced by E-NTPDases and E-NPPs is in turn metabolized to adenosine by ecto-5´-nucleotidase/CD73 [195]. Nucleoside tri, di and monophosphates are equally hydrolysed by alkaline phosphatases including TNAP, which is highly expressed in the CNS [196,197]. In the case of adenosine, this metabolite is generally the product of the ectoenzymatic breakdown of ATP; however, certain neurons and astrocytes are able to release adenosine also directly [198,199]. Adenosine can be removed from the extracellular space by different mechanisms such as its phosphorylation to AMP mediated by adenosine kinase (ADK) or deamination to inosine via the action of adenosine deaminase [200].

## 4. Purinergic Signalling during CNS Development

The early and predominant expression of purinergic receptors and ectonucleotidases in the developing CNS and the capacity of different cells to release ATP gives a cue of the many roles purinergic signalling carries out at the different neurodevelopmental stages. Numerous studies have demonstrated the involvement of purinergic signalling in proliferation, migration and differentiation of neural precursor cells [201,202,203,204]. Likewise, purinergic signalling is also involved in neuronal migration and the subsequent establishment of synaptic contacts as well as synaptogenesis [205,206] processes known to be dysregulated following neonatal seizures [205,206,207,208,209].

### 4.1. Expression and Function of Proteins Involved in Purine Release during Development

During CNS development, several proteins involved in purine release have been described including VNUT, which is expressed by granule cell precursors of the mouse cerebellum [107] and hemichannels such as connexins and pannexins. Regarding connexins, nine members of this family are expressed differentially throughout development [210,211,212,213,214,215], with their expression linked to cell proliferation and migration [211]. Connexins are involved in the regulation of the migration of the neural precursor cells by modulating cell–cell adhesion such as connexin-43, which is located in radial glial fibers [211]. Likewise, the expression pattern of pannexin changes throughout brain development, corresponding these changes with neurogenic and gliogenic processes of embryonic and early postnatal development [95]. Postnatally, Panx1 is expressed by neural and progenitor cells, playing a role in cell proliferation [216]. During CNS development, Panx1 transcripts have been found in the periventricular postnatal neural stem cells (NSCs) and neural progenitor cells (NPCs) [216]. In vitro studies with ventricular zone (VZ)-derived neurospheres have demonstrated that Panx1 is involved in cell proliferation. In line with this, blocking of Panx1 activity with the specific blocker probenecid reduced the proliferative capacity of VZ neurosphere cultures [216]. Moreover, Panx1 mediates the release of ATP, which in turn activates P2 receptors and increases proliferation of NSCs and NPCs [216]. Panx1 has also been linked to cell migration and the control of neurite outgrowth [217]. Panx2, another member of the pannexin family, is expressed in different subsets of neural progenitor cells of the postnatal hippocampus. However, when these cells differentiate into a neuronal lineage, Panx2 expression is downregulated [218].

#### 4.1.1. P1 Receptor Expression and Function during CNS Development

Purinergic receptors are differentially expressed at different stages of embryonic and postnatal neurodevelopment. The expression of P1 purinergic receptors is already detected during embryonic neurodevelopmental stages. The A1 receptor is expressed from E14 and presents a similar allocation to adulthood at E21, being found in the cerebral cortex, hippocampus, thalamus, midbrain and cerebellum of the rat brain [219,220]. Expression of the A2 receptor has been detected from E13 onwards, increasing its expression levels after birth [220,221]. During CNS development, A1 and A2_A_ receptors were involved in processes regulating cell migration, neuronal connectivity and synaptogenesis. Tangential migration of medial ganglionic eminence (MGE)-derived GABAergic interneurons was delayed during pregnancy and lactation periods due to exposure to caffeine, an antagonist of A1 and A2_A_ receptors [221]. The same effect has been observed by using a specific A2_A_ receptor antagonist or A2_A_ receptor knockout mouse pups, demonstrating the involvement of the A2_A_ receptor in the migration of MGE-interneurons [221]. Regarding neuronal connectivity, adenosine receptors may contribute to neurite growth counteractively. In vitro studies have described that activation of the A1 receptor inhibits neurite outgrowth via the Rho-kinase pathway [222]. In contrast, A2_A_ receptor activation promotes the outgrowth of dendrites [223,224] and axonal elongation [224] through different signalling pathways. Finally, the A1 receptor modulates immature neuronal activity in different regions of the brain, including the hippocampus and cortex. In immature CA1 neurons, adenosine inhibits GABA release from the presynaptic nerve terminals through activation of the A1 receptor [225]. Since previous studies have described that A1 receptor activation inhibits glutamatergic release in adult hippocampal neurons [226,227,228,229], these results might confer an additional role to the A1 receptor during development. Moreover, activation of presynaptic A1 receptors inhibits excitatory GABAergic transmission from Cajal–Retzius cells, the early born neurons in layer I of the cortex, to pyramidal neurons in lower cortical layers [230]. Likewise, adenosine can regulate oligodendrogenesis in a bidirectional manner via A1 and A2_A_ receptors. A1 receptor stimulation contributes to maturation and prevents proliferation of the oligodendrocyte precursor cells (OPCs) [231,232]. Conversely, A2_A_ receptor activation inhibits maturation and induces proliferation of OPCs [77].

#### 4.1.2. P2 Receptor Expression and Function during CNS Development

P2X5 is the earliest expressed P2X receptor during development, with P2X5 being detected in mouse neural tubes from E8 and being upregulated to E13. The expression of P2X3 has been detected in mouse neuroectodermal cells [233] and rat brain from E11 onwards [234,235] and its activation induces the proliferation of embryonic stem cells [236]. From E14 onwards, both P2X2 and P2X7 are expressed [235]. Previous data has shown that silencing of the P2X2 receptor promotes proliferation, suggesting that P2X2 regulates this process negatively [237], whereas the P2X7 receptor is expressed in mouse embryonic stem cells and modulates processes involved in proliferation and neural differentiation [238]. The remaining P2X receptors, P2X1, 4 and 6 appear at postnatal stages of rat brain development [235]. P2X1 and P2X3 expression within the brain remains consistent from birth to adulthood, whereas P2X2 expression is downregulated with age. Conversely, neocortical P2X4 and P2X7 expression is upregulated incrementally with age, reaching its peak in adulthood [14]. At P7 age, P2X7 expression is predominately found in microglia and is also expressed in Bergmann glia of the cerebellum [179]. Unfortunately, a definitive answer on neuronal and astrocytic P2X7 expression, not just in infants, is under debate [239]. Multiple groups have observed that neuronal P2X7 localised to presynaptic terminals [240,241]. P2X7 is also expressed in primary neuronal and astrocytic in vitro cultures [242]. However, when using a transgenic P2X7 reporter mouse, in which the green fluorescent protein is fused to the P2X7 receptor, thus allowing visualization of P2X7 expression, neuronal and astrocytic P2X7 is not observed [179,243]. Neuronal and astrocytic P2X7 immunoreactivity was also absent when using a P2X7-specific nanobody in both the immature and adult mouse brain [179]. However, one could hypothesis that neuronal P2X7 expression is below the detection limit with immunoreactivity techniques, is localised to intracellular compartments or is upregulated only in pathology [179,244].

P2Y expression has been mostly located in proliferative regions and at early stages of neurodevelopment. P2Y_1_ expression has been detected from E11 onwards [245], being expressed by radial glial and intermediate precursor cells located in proliferative regions of the developing cortex [201,203]. Neurospheres cultured from the adult subventricular zone (SVZ) exhibited an increase of cell proliferation after P2Y_1_ activation by using several agonists (2-MeSATP, ADPβS, 2-ClATP and 2-MeSADP) [246] suggesting that the P2Y_1_ receptor may be involved in cell proliferation. On the contrary, blocking of P2Y_1_ by the antagonist MRS2179 reduced cell proliferation, and the same effect was observed in P2Y_1_ receptor knockout mice [246]. P2Y_4_ expression has also been detected at E11 stage, being located together with P2Y_2_ in ventricular regions of the E14-telencephalon. Similar to P1 receptors, P2 purinergic receptors present different profiles of location and temporal expression during postnatal stages, suggesting that they also play a role in various stages of brain development [160,205,234,235,245,247,248,249,250].

#### 4.1.3. The Dual Role of the P2X7 Receptor during CNS Development

During CNS development, the P2X7 receptor plays a dual role promoting opposing processes such as cell death and cell proliferation. These opposing effects driven by the same receptor may depend on the cell type that expresses it, the extracellular concentration of ATP or the duration of P2X7 receptor activation. However, the involvement of P2X7 in neuronal cell death is still unclear since there is still controversy about the expression of this receptor in the different cell types of the CNS [11,239], as explained previously.

In mouse embryonic stem cells, the P2X7 receptor promotes its proliferation and maintenance in an undifferentiated state, while for its neural differentiation, P2X7 receptor expression needs to be suppressed [238]. Likewise, the P2X7 receptor is able to induce necrosis of NPCs when activated with high concentrations of ATP or the agonist Bz-ATP [251]. In contrast, stimulation of P2X7 with low concentrations of Bz-ATP leads to neuronal differentiation of NPCs [252]. Moreover, depending on the duration of P2X7 receptor activation, this receptor can mediate pro-survival or pro-death signalling [253]. Additionally, P2X7 might regulate the population of NPCs through innate phagocytosis of dead cells throughout development. In this regard, neuroblasts isolated from human foetal telencephalons are able to phagocytose apoptotic cells in the absence of P2X7 receptor activation [254].

P2X7 has also been identified on microglial cells of the rat brain from late E16 onwards, exhibiting a wide distribution in the forebrain at P30 stage [255]. In line with its known role driving microglia proliferation [181,256], P2X7 has been shown to control microglial proliferation in the embryonic spinal cord of mice at E13.5 stage [257]. Conversely, prolonged P2X7 stimulation with high concentrations of Bz-ATP induces microglia cell death in the cortex of newborn mice [180]. Thus, similar to NPCs, the outcome of P2X7 activation in microglia cells might depend on the amount of available extracellular ATP and the duration of stimulation of the receptor. As such, it can be concluded that P2X7 may act to regulate itself to prevent excessive microglia proliferation during neurodevelopment. Finally, the P2X7 receptor is also expressed on oligodendrocyte progenitors contributing to stimulation of migration and driving oligodendrocyte differentiation [258].

### 4.2. Extracellular Purine Metabolism during Development

The temporal expression of purinergic receptors during brain development is accompanied by modifications in the expression of ectonucleotidases. Individual ectonucleotidase expression varies according to developmental stage and brain region. NTPDase 2, which is the dominant ectonucleotidase expressed by progenitors in the late embryonic and adult mouse brain, has been identified from E18 in neurogenic regions [259,260], whereas NTPDases 1, 3, 5 and 6 are detected in later stages of brain development (P7-21) [261]. Concerning ecto-5´-ectonucleotidase, its expression increases during postnatal stages (it has been identified in migrating neuroblasts of the cerebellum and is related with synaptogenesis processes [192,262,263,264,265]). Certain ectonucleotidases of the E-NNPs family are also expressed at early stages of neural development, such as E-NNP-2, for which the splice variant autotaxin was identified in the floor plate of the neural tube at E9.5 [266]. Postnatally, the expression of E-NNP 1–3 is detected in several regions of the rat brain [267].

Finally, TNAP expression begins at very early stages of neural development, being highly expressed by neuroepithelial stem cells of the neural tube from E8.5 and a migrating subpopulation of neuroectodermal cells [268,269,270]. Moreover, a strong activity of TNAP has been identified in ventricular and subventricular zones, which are high cell proliferative regions, at the E14 stage [260], and postnatally, its activity is related to synaptogenesis in the cerebral cortex [271]. Therefore, TNAP might contribute to cell proliferation or cell differentiation in the neurogenic niche. In NSCs cultured from adult mice, downregulation of TNAP causes a strong decrease in progenitor cell proliferation [272]. In addition, TNAP might be involved in the control of axonal growth during development. Studies with cultured hippocampal neurons have shown that TNAP expressed by outgrowing axons promotes axonal elongation through the hydrolysis of extracellular ATP [273]. As a result of this, extracellular ATP levels are drastically reduced, indirectly modulating activation of purine receptors. Interestingly, TNAP and P2X7 are tightly linked, with the addition of exogenous TNAP increasing P2X7 receptor expression, whereas TNAP expression is downregulated when P2X7 is inhibited. Importantly, TNAP knockout mice exhibit perinatal lethality, with P9 being the maximum reached age [274,275], and present with a decrease in the number of matured cortical synapses and an absence of myelinated cortical axons [276].

In summary, the fundamental role of the purinergic system during neurodevelopment is clear, and with its diverse expression and functionality, there are many avenues to explore that could be effective treatments to early life disorders.

## 5. Purinergic Signalling and Neonatal Seizures

**Figure 2:**
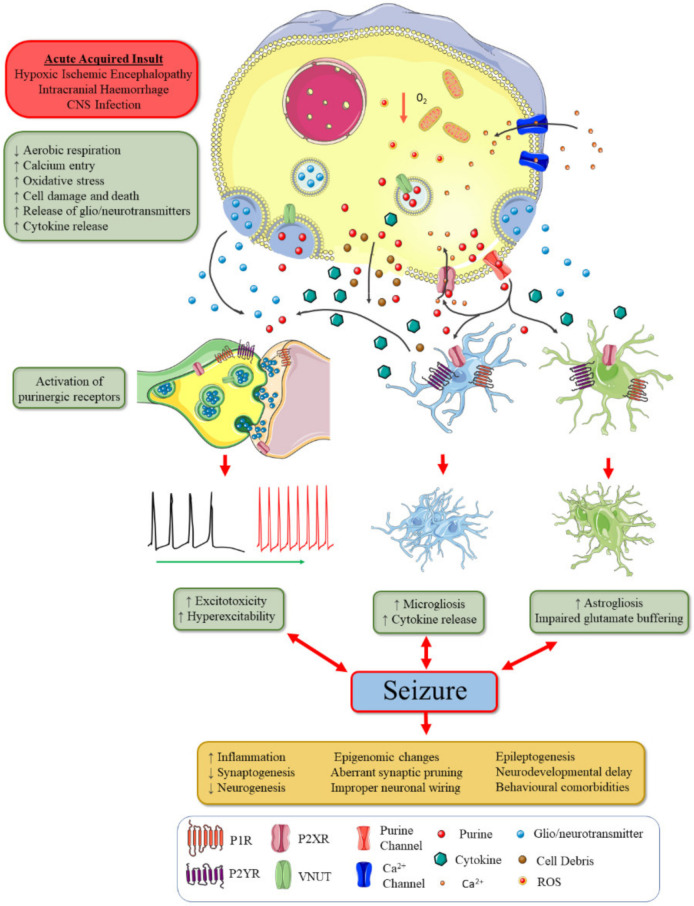
Cellular mechanisms of acute symptomatic neonatal seizure ictogenesis and the potential role of purinergic signalling: following an acute insult to the neonatal brain, cells are placed under high cellular stress, leading to increases in calcium entry and cell death pathways. In the case of hypoxic-ischemic encephalopathy (HIE)-induced seizures, the lack of oxygen and glucoses limits aerobic respiration, forming radical oxygen species (ROS) causing further oxidative stress on cells. Increases in intracellular calcium and cell death can trigger the release of glio/neurotransmitters (e.g., glutamate) into the extracellular space that increases neurotransmission. Cell debris can trigger microgliosis, astrogliosis and release of proconvulsive cytokines. Purines (e.g., ATP and adenosine) are also hypothesised to be released into the extracellular space following cell death and through a combination of exocytotic and non-exocytotic mechanisms under cellular stress. ATP acts upon P2X7 to further increase intracellular calcium, contributing to cell death mechanisms and to increasing neurotransmission and, in turn, seizure severity. P2X7 activation is known to potentiate proconvulsive cytokine release following neonatal seizures, which in turn can lower seizure thresholds. Other P2 receptors are known to modulate many mechanisms of seizure ictogenesis, such as direct modulation of neurotransmission and inflammatory signalling cascades. A2_A_ receptors may also contribute to neonatal seizures via similar mechanism to P2X7. Conversely, A1 receptor activation is anticonvulsive in neonatal seizures, acting as an endogenous compensatory mechanism. Once these outlined mechanisms create a system that favours excitatory neurotransmission, seizures are elicited. A seizure can also create further cellular stress and neuroinflammation, increasing the likelihood of recurrent seizures. Elevated neuroinflammation and hyperexcitability alter many mechanisms critical for brain development, leading to long-lasting changes of the brain. Purinergic signalling can be hypothesised to modulate this and may be targeted in the future to prevent comorbidities following neonatal seizures.

As stated earlier, purinergic signalling is widespread in the immature brain and many studies have targeted this system effectively to modulate seizures in the adult scenario [9]. This section will discuss our current knowledge of how purinergic signalling modulates neonatal seizures and future potential therapeutic avenues to explore (Figure 2). This encompasses studies on both P1 and P2 receptors. An overview of studies investigating neonatal seizures and the purinergic system is displayed in Table 1.

### 5.1. Targeting of P1 Receptors

As early as 1988, when the purinergic signalling field was in its infancy, the nucleoside adenosine was proposed as an endogenous anticonvulsant [277]. When adenosine is applied to resected epileptic hippocampal slices, it was shown to arrest epileptiform activity [278]. In fact, adenosine is released into the brain following seizures of temporal lobe epilepsy patients, where it may act as an endogenous mechanism to arrest seizures [278], whereas caffeine, a nonspecific adenosine receptor antagonist, acts as a convulsant compound, potentiating the seizure phenotype following PTZ injection [279,280]. Various case reports also show caffeine to induce seizures in non-epileptic persons [281].

Currently, there is only evidence of A1 and A2_A_ receptors modulating seizure phenotypes. Pometlova et al., 2010, showed the potential of targeting the P1 receptors, with the nonspecific adenosine receptor agonist, 2-chloradenosine, having an anticonvulsive effect in immature rats following cortical stimulation [282]. These effects were not model-specific, with PTZ-induced seizures in immature rats being suppressed by 2-chloradenosine administration [283]. Building upon this, using specific agonists and antagonists of A1 and A2_A_ receptors, Mares observed that the anticonvulsive effects seen was primarily due to action upon A1 receptors, with pharmacological targeting of A2_A_ having little effect in P12 rats, the age that relates most to a human neonate [283]. Anticonvulsant action of A1 receptors was reinforced with agonistic action reducing the magnitude of elicited cortical discharges [284]. Again, this effect was more pronounced in P12 rats rather than P25, suggesting a possible developmental shift in the sensitivity of adenosine receptors [284]. Interestingly, in this model, both agonistic and antagonistic action of A2_A_ receptors had an anticonvulsive effect in P12 rats, yet blocking A2_A_ receptors in P25 produces a proconvulsive effect [284]. These results were replicated, even when a different area of the brain (hippocampus) was stimulated to induce seizures [285], with the A1 receptor agonist 2-chloro-*N*6-cyclopentyladenosine having an anticonvulsive effect at all ages except P25. These studies highlight the developmental regulation of P1 receptors and the possible age-dependent modulation of seizure phenotypes. Altered expression levels of adenosine receptors has been observed 48 h following induced febrile seizures in neonatal rats, with the A1 receptor increasing and the A2_A_ receptor decreasing [286]. This suggests that the adenosine system may act as endogenous compensatory mechanism for seizures. Currently, there are limited studies investigating the anti-epileptogenic capacity of targeting P1 receptors. Possible adverse effects of modulation of the adenosine system have been unexplored in these neonatal seizures studies, yet the authors acknowledge the necessity for this. Likewise, only the effect of caffeine, an A1 antagonist, on neurodevelopment has been studied. Caffeine is shown to ameliorate phenobarbital impairment of neurogenesis in neonatal rats [287], possibly due to caffeine’s ability to suppress GABAergic action [288]. However, when it is given in isolation, caffeine reduces the proliferative capacity of the brain [287]. In the adult mouse, caffeine can reduce long-term potentiation and can alter synaptic plasticity, which could be detrimental in the immature brain [289]. One such rodent study shows that early life exposure to caffeine can increase the seizure susceptibility in adulthood [290]. Conversely, at low doses, caffeine may act to reduce acute seizures, particularly in the infant brain, with neonatal rats having an increased seizure threshold to chemoconvulsants following a low dose of caffeine [291]. Interestingly, many studies have shown a neuroprotective effect of a low dose of caffeine in the setting of HIE, reducing white matter injury and protecting against memory impairment [292] and motor deficits in later life [293]. These studies highlight the complex nature of early life seizures and how mechanisms of seizure ictogenesis may differ from epileptogenesis.

Despite the presence of adenosine signalling in the majority of biological systems, little is known about the adverse effects of adenosine receptors in the CNS and concerns for unwanted side effects are well warranted. With P1 receptors having a large role in cardiovascular and respiratory function via action upon the brainstem, the sudden rise of endogenous adenosine following seizures is hypothesised as one contributing factor to Sudden Unexpected Death in Epilepsy (SUDEP) [294]. Also, despite the documented use of many P1 receptor ligands reported in the literature, only adenosine and regadenoson (A2_A_ receptor antagonist) are approved for use in the clinic [295].

### 5.2. Targeting of P2 Receptors

Of the P2 receptors, targeting of the P2X7 receptor has shown the most promise in neonatal seizures. A role for the P2X7 receptor in seizures was first examined in adult seizures, where using transgenic and pharmacological tools showed it to have a proconvulsive or anticonvulsive action depending on experimental model used [244].

Mesuret et al., 2014, first investigated the P2X7 receptor in the neonatal seizure scenario. P2X7 receptor expression was upregulated as early as one hour in the hippocampus following seizures induced via intra-amygdala injection of KA in P10 rats. P2X7 expression increased to a maximum at 72 h post-KA that was also accompanied by elevated levels of the cytokine IL-β [40]. Interestingly, treatment with the P2X7 antagonist A-438079 reduced the acute electrographic seizures by over 50%, was neuroprotective and reduced levels of seizure-induced neuroinflammation. Importantly, treatment with A-438079 had greater neuroprotective effects than treatment with current clinical used drugs, phenobarbital and bumetanide. In fact, phenobarbital and bumetanide failed to show any neuroprotective effects [40]. These results have been translated in a model more clinically relevant. Using global hypoxia to induce seizures (5% O_2_, 15 min), P2X7 receptor expression was increased 24 h post-seizure. Interestingly, P2X4 receptor expression was also increased 24 h post-seizures suggesting a new avenue to explore. More importantly, P2X7 receptor protein levels were elevated in human infant brain tissue 3 months after a HIE/seizure event [14]. Two different P2X7 antagonists, A-438079 and JNJ-47965567, were able to reduce hypoxia-induced electrographic seizures in neonatal mouse pups. Antagonistic action was also able to reduce levels of pro-inflammatory markers (e.g., IL-1β) 24 h post-seizures. The limitation of these two studies is that P2X7 receptor antagonists were given before seizures ictogenesis, which is not clinically viable. In addition, as P2X7 receptor antagonism reduced the acute insult, it cannot be concluded that P2X7 receptor antagonism alone is able to reduce post-seizure inflammation. However, this is the most likely case due to the major role of P2X7 in pathological inflammation. With inflammation heavily involved in pathology following neonatal seizures, the P2X7 receptor might have a role in epileptogenesis following an insult to the infant brain. Further investigation with post-seizure treatment to investigate the ability of P2X7 to prevent neonatal seizure comorbidities is much anticipated. As aforementioned, more P2 receptors are currently being explored in adult seizures, whereas now, only P2X7 has been targeted in neonatal seizures. A further therapeutic target requiring further investigation is P2X4, with its expression upregulated following neonatal seizures [14]. Under hypoxic conditions (5% O_2_, 3.5 h, P0), P2X4 was again upregulated in immature rats. Furthermore, this upregulation was greater than that observed with P2X7 and P2Y_12_ [296]. P2X4 is described to mediate ATP-gated microglia activation and release of proconvulsive inflammatory cytokines in these hypoxic conditions [296]. Again, with inflammation heavily involved in seizure ictogenesis and epileptogenesis, one could hypothesis targeting P2X4 to be advantageous for the treatment of neonatal seizures.

### 5.3. Potential Purinergic Targets to Explore

Finally, apart from direct action upon membrane-bound receptors, another strategy to explore would be to regulate concentrations in the extracellular space of purine nucleotides and nucleosides. This can be achieved via inhibition of enzymes, such as ADK, to reduce the clearance of adenosine. Pharmacological and genetic evidence shows that ADK has a role in adult epilepsy development and seizure generation [297,298,299]. Hypophosphatasia, in which neonatal seizures are a major component, is heavily associated with mutations in the TNAP gene. In fact, mice deficient in TNAP show spontaneous seizures by P6 [274,275]. Interestingly, TNAP-related seizures are mediated via P2X7. Mice double deficient in TNAP and P2X7, as well as TNAP knock-out mice treated with a P2X7 receptor antagonist, did not present with spontaneous seizures [274]. Furthermore, antagonistic action against TNAP increased the seizure duration in adults, and thus, it would be interesting to investigate TNAP in neonatal seizure models [274]. With TNAP’s roles in regulating synaptic function during neuronal development [300], targeting TNAP in the neonatal seizure scenario could aid in preventing the comorbidities seen in this condition.

Apart from regulating the metabolism of purines, one potential strategy would be to prevent the release of ATP into the extracellular space. VNUT is a relatively unexplored target in relation to seizure modulation yet, with its prominent role in ATP release, is an exciting avenue to explore. With its prominent expression in the immature brain, targeting Panx1 may also show promise in modulating neonatal seizures. Panx1 is shown to be active in KA-induced seizures in juvenile mice (P13–14) which corresponds with a doubling in extracellular ATP levels [301]. In Panx1 null mice and when Panx1 was blocked with pharmacological tools, behavioural seizure manifestations were reduced [301]. Interestingly, Panx1 seems to not be involved in seizure ictogenesis yet is involved in maintaining seizure activity. It would be interesting to see if this result can be translated to an age more appropriate to neonatal seizures.

As stated earlier, there are many components of the purinergic system that are present early on during development, with the majority unexplored in the role of seizure generation and epileptogenesis. It would be advantageous to investigate these in further detail to uncover the full picture of purinergic system in neonatal seizures, to maximise the efficiency of future pharmacological drugs and to minimise adverse effects. Currently, no study examines purinergic signalling away from the initial neonatal seizure event. In the clinic, it may prove difficult to prevent the initial neonatal seizure, and further investigation into preventing further recurrent seizures is needed. Purinergic signalling is involved in many processes known to contribute to epileptogenesis and to potentiate damage. Targeting inflammation following neonatal seizures, a process in which purinergic signalling is heavily involved, has shown promise to reduce the development of epilepsy and behavioural deficits [302].

## 6. Conclusions

Current therapies for neonatal seizures seemed to be limited to direct modulation of ion channels on neurones. As we have progressed in understanding seizure pathology, we now know that many mechanisms, such as chronic neuroinflammation, blood–brain barrier dysfunction and aberrant neurogenesis, can influence seizure ictogenesis. This allows us to use many more potential mechanisms to target greater efficacy. As outlined in this review, the purinergic system is widely expressed within the CNS and has a multitude of physiological and pathological functions. We are still lacking knowledge in many aspects of what role the purinergic system has in contributing to neonatal seizure pathology, but studies have shown great promise in targeting this biological system, particularly in targeting the P2X7 receptor. Further studies are needed not only in uncovering mechanisms of how purinergic signalling may influence neonatal seizures and subsequent pathologies but also in investigating the fundamental mechanisms of neonatal seizure pathology itself.

## Figures and Tables

**Table 1:** Overview of studies investigating purinergic signalling modulating neonatal seizures.

Target Receptor	Compound	Seizure Model	Species, Age and Gender	Effect	Reference
**P1**					
Nonspecific P1	2-chloroadenosine (1, 4 and 10 mg/kg, i.p) (agonist)	Cortical epileptic after discharges (drug administered 5 min first after discharge)	Rats (P12, P18 and P25); sex not specified	Behavioural and EEG-detected seizures were only reduced at P18.	[282]
Nonspecific P1	2-chloroadenosine (1, 5, 10 and 15 mg/kg, i.p.) (agonist)	PTZ 100 mg/kg s.c. (90 mg/kg in P18). (drugs were administered 30 min before seizure induction)	Rats (P7, P12, P18, P25 and P90); males	Anticonvulsive effect was seen at all ages. Suppression of tonic seizures was only at P12 and younger. Suppression of generalised seizures was at P18 and above.	[283]
A1	2-chloro-*N*6-cyclopentyladenosine (0.2, 0.5 and 1 mg/kg to 12-day-old rats and 0.5, 1 and 2 mg/kg to 25-day-old rats, i.p) (agonist)		Rats (P12 and P25); males	2-chloro-*N*6-cyclopentyladenosine led to marked anticonvulsant effects in P12. Minimal effects were seen in P25. No effect was seen with DPCPX.	
	DPCPX (1 and 2 mg/kg i.p.) (Antagonist)				
A2A	CGS 21680 (0.1, 0.2, 0.5, 1, 2 and 5 mg/kg, i.p.) (agonist)			Highest dose of CGS 21680 (5mg/kg) reduced seizure severity only at P25. No effect was observed in P12 at any dose. No effect was observed with ZM 241385.	
	ZM 241385 (1, 2 and 5 mg/kg, i.p.) (antagonist)				
A1	2-chloro-*N*6-cyclopentyladenosine (0.5) and 1 mg/kg i.p.) (agonist)	Cortical epileptic after discharges (drugs were administered 5 min after first stimulation)	Rats (P12, P18 and P25); males	Duration reduced after discharges with agonist and proconvulsant action of antagonist at P12 and P18. At P25, both agonistic and antagonistic action are proconvulsive.	[284]
	DPCPX (1 and 2 mg/kg, i.p.) (antagonist)				
A2A	CGS 21680 (0.5 and 5 mg/kg i.p.) (Agonist)			CGS 21680 is anticonvulsive at all ages. While ZM 241385 action is anticonvulsive at P12 and P18, it is proconvulsive at P25 at the highest dose.	
	ZM 241385 (1 and 5 mg/kg i.p.) (antagonist)				
Nonspecific P1	2-chloro-*N*6-cyclopentyladenosine (0.5 and 1 mg/kg, i.p) (agonist)	Hippocampal epileptic after discharges. (drug administered 10 min prior to the stimulation procedure)	Rats (P12–P60); males	Anticonvulsive effect was seen in all ages bar P25. Hippocampal A1 protein expression peaks at P10 and decreases with age.	[285]
**P2**					
P2X7	A-438079 (5 and 15 mg/kg, i.p) (antagonist)	Intra-amygdala KA (2 µg in 0.2 µL PBS) (drug administered 1 h post-KA injection)	Rats (P10); mixed sex group	A-438079 reduced seizure severity, subsequent neuronal damage and inflammation.	[40]
P2X7	A-438079 0.5, 5, 15, 25 and 50 mg/kg, i.p.) (antagonist)	Global hypoxia (5% O_2_ 15 min) (drugs administered 5 min prior to hypoxia)	Mice (P7); mixed sex group	P2X7 expression is increased 24 h following hypoxia-induced seizures in the hippocampus. P2X7 expression increased in tissue from patients who experienced HIE and seizures. Both compounds reduced seizure severity. A-438079 reduced post-seizure inflammation.	[14]
	JNJ-47965567 (10 and 30 mg/kg, i.p.) (antagonist)				

Abbreviations: DPCPX, 8-Cyclopentyl-1,3-dipropylxanthine; EEG, electroencephalogram; HIE, hypoxia-ischemia encephalopathy, i.p., intraperitoneal; KA, kainic acid; s.c. subcutaneous; PTZ, Pentylenetetrazole.

## Abbreviations

ADK	Adenosine kinase
Ado	Adenosine
ADP	Adenosine diphosphate
AMP	Adenosine monophosphate
ASD	Antiseizure drugs
ATP	Adenosine triphosphate
cAMP	Cyclic AMP
CNS	Central nervous system
CNTs	Concentrative nucleoside transporters
EEG	Electroencephalogram
E-NPPs	Ectonucleotide pyrophosphatase and/or phosphodiesterases
E-NTPDases	Ectonucleoside triphosphate diphosphohydrolases
ENTs	Equilibrative nucleoside transporters
GABA	γ-aminobutyric acid
HIE	Hypoxic-ischemic encephalopathy
IL-1β	Interleukin-1β
KA	Kainic acid
MCAO	Medial carotid artery occlusion
MGE	Medial ganglionic eminence
NAD^+^	Nicotinamide adenine dinucleotide
NMDA	*N*-methyl-d-aspartate receptor
NPCs	Neural progenitor cells
NSCs	Neural stem cells
NTs	Nucleotides
OPCs	Oligodendrocyte precursor cells
Panx	Pannexin
PTZ	Pentylenetetrazole
SVZ	Subventricular zone
TNAP	Tissue nonspecific alkaline phosphatase
UDP	Uridine monophosphate
UTP	Uridine triphosphate
VNUT	Vesicular nucleotide transporter
VZ	Ventricular zone
